# Sustained hyperammonemia induces TNF-a IN Purkinje neurons by activating the TNFR1-NF-κB pathway

**DOI:** 10.1186/s12974-020-01746-z

**Published:** 2020-02-22

**Authors:** Tiziano Balzano, Yaiza M. Arenas, Sherry Dadsetan, Jerónimo Forteza, Sara Gil-Perotin, Laura Cubas-Nuñez, Bonaventura Casanova, Francisco Gracià, Natalia Varela-Andrés, Carmina Montoliu, Marta Llansola, Vicente Felipo

**Affiliations:** 1 Laboratory of Neurobiology, Centro Investigación Príncipe Felipe, Eduardo Primo-Yufera 3, 46012 Valencia, Spain; 2 Instituto Valenciano de Patología, Unidad Mixta de Patología Molecular, Centro Investigación Príncipe Felipe/Universidad Católica de Valencia, Valencia, Spain; 3 Multiple Sclerosis and Neuroimmunology Research Group, Fundación para la Investigación La Fe, Valencia, Spain; 4 Instituto de Investigacion Sanitaria INCLIVA, Hospital Clinico de Valencia, Valencia, Spain

**Keywords:** Hyperammonemia, Neuroinflammation, TNF-a, Purkinje neurons, TNFR1

## Abstract

**Background:**

Patients with liver cirrhosis may develop hepatic encephalopathy. Rats with chronic hyperammonemia exhibit neurological alterations mediated by peripheral inflammation and neuroinflammation. Motor incoordination is due to increased TNF-a levels and activation of its receptor TNFR1 in the cerebellum.

The aims were to assess (a) whether peripheral inflammation is responsible for TNF-a induction in hyperammonemic rats, (b) the cell type(s) in which TNF-a is increased, (c) whether this increase is associated with increased nuclear NF-κB and TNFR1 activation, (d) the time course of TNF-a induction, and (e) if TNF-a is induced in the Purkinje neurons of patients who die with liver cirrhosis.

**Methods:**

We analyzed the level of TNF-a mRNA and NF-κB in microglia, astrocytes, and Purkinje neurons in the cerebellum after 1, 2, and 4 weeks of hyperammonemia. We assessed whether preventing peripheral inflammation by administering an anti-TNF-a antibody prevents TNF-a induction. We tested whether TNF-a induction is reversed by R7050, which inhibits the TNFR1-NF-κB pathway, in ex vivo cerebellar slices.

**Results:**

Hyperammonemia induced microglial and astrocyte activation at 1 week. This was followed by TNF-a induction in both glial cell types at 2 weeks and in Purkinje neurons at 4 weeks. The level of TNF-a mRNA increased in parallel with the TNF-a protein level, indicating that TNF-a was synthesized in Purkinje cells. This increase was associated with increased NF-κB nuclear translocation. The nuclear translocation of NF-κB and the increase in TNF-a were reversed by R7050, indicating that they were mediated by the activation of TNFR1. Preventing peripheral inflammation with an anti-TNF-a antibody prevents TNF-a induction.

**Conclusion:**

Sustained (4 weeks) but not short-term hyperammonemia induces TNF-a in Purkinje neurons in rats. This is mediated by peripheral inflammation. TNF-a is also increased in the Purkinje neurons of patients who die with liver cirrhosis. The results suggest that hyperammonemia induces TNF-a in glial cells and that TNF-a released by glial cells activates TNFR1 in Purkinje neurons, leading to NF-κB nuclear translocation and the induction of TNF-a expression, which may contribute to the neurological alterations observed in hyperammonemia and hepatic encephalopathy.

## Background

Patients with liver cirrhosis may develop minimal hepatic encephalopathy (MHE) with mild cognitive impairment, attention deficits, motor incoordination, and psychomotor slowing, which impair quality of life, reduce the lifespan, and increase accidents, falls, and hospitalizations. MHE affects several million people and is a serious health, social, and economic problem [1].

Hyperammonemia and peripheral inflammation play synergistic roles in inducing cognitive and motor alterations in MHE [2–5].

Studies in animal models of hyperammonemia and MHE have shown that the emergence of neurological alterations is mediated by the induction of neuroinflammation, which alters glutamatergic and GABAergic neurotransmission, leading to cognitive and motor impairment [5–15]. Both in patients and in animal models, the cerebellum seems to be the first brain area to be affected in early stages of chronic liver diseases [6, 16–19]. Neuroinflammation is more severe in the cerebellum than in other brain areas in animal models [6] and is also prominent in the cerebella of patients with chronic liver disease, even in the steatohepatitis stage, before liver cirrhosis develops [20, 21].

In agreement with the early emergence of neuroinflammation in the cerebellum, one of the earliest alterations in hyperammonemia and MHE is the impairment of motor coordination, which is modulated mainly by GABAergic neurotransmission in the cerebellum. We have recently shown that in hyperammonemic rats, motor incoordination is a consequence of increased TNF-a levels and activation of its receptor TNFR1 in the cerebellum, which increases GABAergic neurotransmission. The administration of extracellular cGMP normalizes the activation of TNFR1, GABAergic neurotransmission, and motor coordination in hyperammonemic rats [13].

However, it remains unclear how hyperammonemia induces an increase in TNF-a in the cerebellum and which cell types show increased TNF-a content.

We recently showed that hyperammonemia per se induces peripheral inflammation, which is the main contributor to neuroinflammation, in the hippocampus and to cognitive impairment [22]. Sustained peripheral inflammation also leads to cognitive impairment in other pathological situations, such as in rheumatoid arthritis, experimental autoimmune neuritis, and diabetes and after major surgery [23, 24]. Reducing peripheral inflammation by the administration of an anti-TNF-a antibody improves cognitive function in patients with chronic inflammatory diseases such as rheumatoid arthritis and sarcoidosis [25, 26] and in animal models of MHE [9, 10, 22].

It is therefore likely that hyperammonemia-induced peripheral inflammation is the trigger of TNF-a induction in the cerebellum of rats. The first aim of this study was to assess whether peripheral inflammation is responsible for the induction of neuroinflammation and TNF-a in the cerebella of hyperammonemic rats. To answer this question, we assessed whether preventing peripheral inflammation by treating rats with an anti-TNF-a antibody (infliximab) prevents neuroinflammation and TNF-a induction in hyperammonemic rats.

In the central nervous system, microglia and astrocytes are usually the main sources of TNF-a. However, a few studies have shown that TNF-a synthesis is induced in neurons in some pathological situations, including stroke [27], intracerebral hemorrhage [28], spinal cord injury [29], and sciatic nerve injury [30]. TNF-a expression is also induced in neurons in the hippocampi of rats with hepatic encephalopathy [10]. The second aim of this work was to assess the cell type(s) in which TNF-a is increased in the cerebella of hyperammonemic rats.

Over the course of the study, we found that sustained hyperammonemia induced the expression of TNF-a in Purkinje neurons.

NF-κB is a major transcription factor that controls the expression of many genes, is involved in multiple aspects of innate and adaptive immune responses, inducing the expression of many proinflammatory genes, including TNF-a [31, 32], and participates in inflammasome regulation [33].

In resting cells, NF-κB dimers (predominantly p50/p65 complexes) are retained in the cytoplasm by binding to IkB. The activation of the IkB kinase (IKK) complex leads to the phosphorylation of IkB, dissociation of NF-κB, and translocation of NF-κB to the nucleus; NF-κB binds to specific DNA sequences and promotes the transcription of target genes, including TNF-a [34]. We assessed whether the induction of TNF-a by hyperammonemia is associated with increased nuclear expression of NF-κB and how hyperammonemia activates NF-κB.

The activation of NF-κB may be induced by the activation of different receptors, including the TNF-a receptor TNFR1. As we have previously shown that the activation of TNFR1 is increased in the cerebella of hyperammonemic rats [13], we assessed whether the activation of NF-κB and induction of TNF-a in Purkinje neurons is mediated by the activation of TNFR1.

Following TNFR1 stimulation, the intracellular death domain (DD) recruits RIP1 and TNFR-associated death domain (TRADD). TRADD engages TNFR-associated factor 2 (TRAF2), inhibitor of apoptosis protein 1 (cIAP1), and inhibitor of apoptosis protein 2 (cIAP2), leading to the formation of complex I [35], which activates IκB kinase, allowing the translocation of NF-κB to the nucleus [36, 37].

To assess whether the activation of NF-κB and induction of TNF-a expression in Purkinje neurons is mediated by the activation of TNFR1, we assessed whether these processes are prevented by R7050. R7050 does not affect the binding of TNF-a to TNFR1 but selectively inhibits the association of TNFR1 with intracellular adaptor molecules (e.g., TRADD and RIP) and thus inhibits subsequent cellular responses after TNF-a binding [38].

We also assessed whether TNF-a is induced in the Purkinje neurons of patients who die with liver cirrhosis and have suffered sustained hyperammonemia.

Therefore, the aims of this work were as follows:

1. To assess whether peripheral inflammation is responsible for the induction of neuroinflammation and TNF-a in the cerebella of hyperammonemic rats.
2. To assess the cell type(s) in which TNF-a is increased in the cerebella of hyperammonemic rats.
3. To provide some insights into the mechanisms by which sustained hyperammonemia induces TNF-a in Purkinje neurons to assess whether (a) the induction of TNF-a by hyperammonemia is associated with increased nuclear expression of NF-κB or (b) the activation of NF-κB and induction of TNF-a in Purkinje neurons are mediated by the activation of TNFR1.
4. To analyze the time course of TNF-a induction in Purkinje neurons and glial cells following the induction of hyperammonemia.
5. To assess whether TNF-a is also induced in the Purkinje neurons of patients who die with liver cirrhosis.

## Material and methods

### Postmortem human samples

Formalin-fixed paraffin-embedded sections (5-μm thick) from the cerebella of 4 patients who died with liver cirrhosis and 3 matched controls (subjects with no liver or neurodegenerative diseases) were obtained from the Instituto de Medicina Legal y Ciencias Forenses (Valencia), Hospital Universitario Fundación Alcorcon and A Coruña Biobank. For each case, appropriate authorization to collect the tissue for research was obtained. The characteristics of the subjects, cause of death, and postmortem delay are detailed in Table 1.

**Table 1:** Characteristics and sources of the human samples

Case	Grade	Sex	Age	Cause of death	PMD (h)	Biobank
Case 20	Control	M	62	Mesenteric ischemia	14	FHU
Case 21	Control	M	72	N.A.	18	FHU
Case 22	Control	M	60	Bronchoaspiration	N.A.	FHU
Case 4	Cirrhosis	M	53	Gastrointestinal bleeding	12–24	IML
Case 8	Cirrhosis	M	69	Gastrointestinal bleeding	17	IML
Case 15A51	Cirrhosis	N.A.	N.A.	Intra-abdominal sepsis	N.A.	A Coruña Biobank
Case 13A37	Cirrhosis	M	54	Metabolic coma	N.A.	A Coruña Biobank

*FHU* Hospital Universitario Fundación Alcorcon, *IML* Instituto de Medicina Legal y Ciencias Forenses (Valencia), *PMD* postmortem delay, *N.A*. data not available

### Rats

**Fig. 1:**
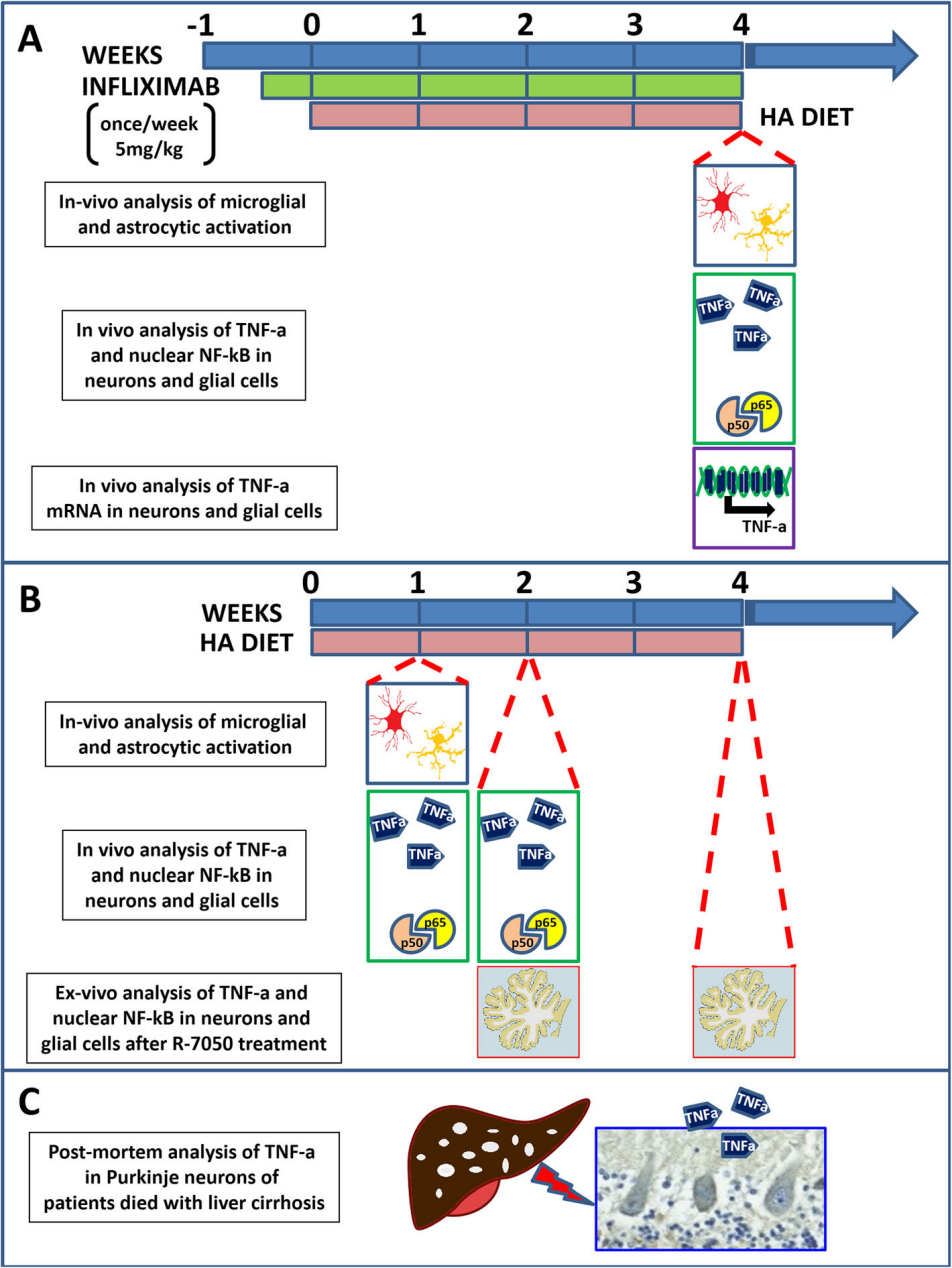
Experimental design. **a** Rats were fed an ammonia-containing diet for 4 weeks and sacrificed by perfusion for in vivo immunohistochemical analysis of microglial and astrocyte activation (blue box), the protein expression of nuclear NF-kB and TNF-a in glia and Purkinje neurons (green box), and the mRNA expression of TNF-a in glia and Purkinje neurons (violet box) in the cerebellum. The effects of peripheral intravenous infliximab administration were assessed by injecting the drug into the rats once per week (5 mg/kg) starting 3 days before the administration of an ammonia-containing diet. **b** Rats were fed an ammonia-containing diet and sacrificed at different time points for in vivo analysis of microglial and astrocyte activation after 1 week of hyperammonemia (blue box), in vivo analysis of nuclear NF-kB and TNF-a protein expression in glia and Purkinje neurons after 1 and 2 weeks of hyperammonemia (green box), and ex vivo analysis of nuclear NF-kB and TNF-a protein expression in glia and Purkinje neurons after 2 and 4 weeks of hyperammonemia (red box). **c** TNF-a expression in Purkinje neurons was also analyzed by immunohistochemistry on postmortem cerebellar samples from patients who died with liver cirrhosis

Male Wistar rats were made hyperammonemic by feeding them an ammonium-containing diet as previously described [39]. The experiments were approved by the Comite de Experimentación y Bienestar Animal (CEBA) of our center and by the Conselleria de Agricultura of Generalitat Valenciana and were performed in accordance with the guidelines of the Directive of the European Commission (2010/63/EU) for the care and management of experimental animals. The experimental design including the 3 different types of experiments performed is summarized in Fig. 1.

### Effects of chronic hyperammonemia and peripheral anti-TNF-a antibody treatment on TNF-a expression in cerebellar microglia, astrocytes, and Purkinje neurons

The rats were randomly distributed into four groups: the control, control+infliximab, hyperammonemic, and hyperammonemic+infliximab group. Infliximab is an anti-TNF-a antibody that does not cross the blood-brain barrier [40], binds to peripheral TNF-a, and reduces peripheral inflammation in rats with MHE or hyperammonemia [9, 10, 22]. Infliximab (Remicade; Merck Sharp & Dohme, Spain) was dissolved in saline (0.9% sodium chloride) and administered by i.v. injection (5 mg/kg) in the tail vein as described previously [41]. The first administration of infliximab was performed 2 days before the start of the ammonium-containing diet, and injections were repeated weekly for 4 weeks. Control rats were injected i.v. with saline. At the end of the 4th week, the rats were sacrificed by perfusion for in vivo immunohistochemical analysis of microglial and astrocyte activation (blue box), the protein expression of nuclear NF-kB and TNF-a in glia and Purkinje neurons (green box), and the mRNA expression of TNF-a in glia and Purkinje neurons (violet box) in the cerebellum (Fig. 1a).

### Time course of TNF-a expression and the role of the TNFR1-NF-κB pathway in hyperammonemic rats

Rats were fed an ammonium-containing diet as described above and sacrificed at different times (1, 2, or 4 weeks). In vivo analysis of microglial and astrocyte activation was performed after 1 week of hyperammonemia (blue box), and in vivo analysis of nuclear NF-kB and TNF-a protein expression in glia and Purkinje neurons was performed after 1 and 2 weeks of hyperammonemia (green box) (Fig. 1b). To study the role of the TNFR1-NF-κB pathway in TNF-a synthesis, untreated and R7050-treated cerebellar slices were used for immunohistochemical analysis of nuclear NF-kB and TNF-a protein expression in glia and Purkinje neurons after 2 and 4 weeks of hyperammonemia (red box) (Fig. 1b).

### Immunohistochemistry

Rats were anesthetized with sodium pentobarbital and transcardially perfused with 0.9% saline followed by 4% paraformaldehyde in 0.1 M phosphate buffer (pH 7.4). The brains were removed and postfixed in the same fixative solution for 24 h at 4 °C. Paraffin-embedded sections (5-μm thick) from the rats or humans were cut and mounted on coated glass slides, processed with the Envision Flex+ Kit (Dako) to block endogenous peroxidase activity for 5 min, and incubated with an anti-Iba-1 (1:300; 30 min; Wako), anti-CD-68 (1:100; 60 min; Abcam), anti-GFAP (ready-to-use; 20 min; Dako), or anti-TNF-a (1:2000; 45 min; Abcam) antibody. The reaction was visualized by incubation with Envision Flex+ horseradish peroxidase for 20 min and then with diaminobenzidine for 10 min. The sections were counterstained with Mayer’s hematoxylin for 5 min.

### Analysis of astrocyte and microglial activation

Analysis of Iba-1, CD68, and GFAP staining in the white matter of the cerebellum was performed using ImageJ software. Brain sections from six animals per group were used. Microglial activation was assessed by measuring the perimeter of Iba-1-stained cells in 10 randomly selected 56x fields per section. The results are expressed in micrometers. To further assess microglial activation, staining for CD68, a marker of activated microglia [42], was analyzed. The number of CD68^+^ cells was counted using the cell counter function of ImageJ, and the results are expressed as cells/mm^2^.

For GFAP quantification, the area of interest was selected. Using the auto local threshold and analyze particle functions, intensity thresholds and size filters were applied. To measure the total amount of GFAP, no size filter was applied. For each rat, at least 10 56x fields were quantified. The results are expressed as the percentage of the area stained with GFAP.

### Analysis of TNF-a content in the white matter and Purkinje layer

Analysis of TNF-a staining in the white matter and Purkinje layer of the cerebellum was performed using ImageJ software. The number of cells expressing TNF-a in the white matter of the cerebellum was manually counted using the cell counter plugin of ImageJ, and the results are expressed as cells/mm^2^. For the analysis of Purkinje neurons, this region was manually selected using the freehand selection tool of the ROI manager function, and the mean intensity (M.I.) of TNF-a staining was recorded. Analysis of each region was performed in at least 10 56x fields (for the white matter) and 10 40x fields (for the Purkinje layer) from each rat or subject.

### Analysis of TNF-a expression in patients who died with liver cirrhosis

Formalin-fixed paraffin-embedded sections from the cerebella of 4 cirrhotic patients and 3 control subjects were used for the immunohistochemical analysis of TNF-a expression in human Purkinje neurons as described above (Fig. 1c).

*Fluorescence in situ hybridization* was performed to detect TNF-a mRNA expression in 5-μm cerebellar sections as previously described [10]. In brief, slices were deparaffinized and rehydrated, and the tissue was digested with proteinase K (Ambion-Life Technologies). A fluorescein-conjugated 23-nucleotide probe (50 μM; Exiqon) was diluted in hybridization solution (50 ng/μl) with 30% formamide and denatured at 80 °C for 2 min. The slices were incubated for 16 h in a humidified hybridization chamber at 60 °C. The next day, two stringency washes were performed, one with 1X SSC at 48 °C for 15 min and one with 1X SSC at room temperature for 15 min. The slices were counterstained with 4′,6-diamidino-2-phenylindole (DAPI; 5 μg/ml; Sigma-Aldrich). The slices were observed under a confocal microscope and imaged. To quantify the content of TNF-a mRNA in Purkinje neurons, cells were manually outlined using ImageJ, and the mean intensity (M.I.) was measured. For the white matter, the number of cells expressing TNF-a was manually counted using the cell counter plugin of ImageJ, and the results are expressed as cells/mm^2^.

Double immunofluorescence staining was performed to confirm the localization of TNF-a in microglia (using Iba-1; 1:300; Abcam), astrocytes (using GFAP; 1:400; Sigma-Aldrich), and Purkinje neurons (using Calbindin; 1:200; Abcam).

### Immunofluorescence analysis of the subcellular distribution of NF-κB p50 and p65

Analysis of the p50 and p65 subunits of NF-κB was performed by immunofluorescence. Sections from six different animals from each group were selected, washed in 0.1 M phosphate buffer, and blocked with normal serum from the same species as the secondary antibody before being incubated overnight with primary antibodies (NF-κB p50 (1:200), NF-κB p65 (1:100), and Iba-1 (1:300); Abcam; GFAP (1:400); Sigma-Aldrich) diluted in blocking buffer and fluorescent secondary antibodies (1:400; Invitrogen). The nuclei were counterstained with DAPI (Sigma-Aldrich), and the sections were coverslipped. The sections were observed under a confocal microscope (Leica TCS-SP2-AOBS) and imaged.

The p50 and p65 subunits may be located in the nucleus, nucleolus, or cytosol. The nuclear, nucleolar, and cytoplasmic intensities of the p50 and p65 subunits were analyzed using ImageJ (1.48v). Nuclei and nucleoli were outlined on the blue (DAPI) channel using the ROI manager function, and the selection was applied on the green channel (p50 or p65) to measure fluorescence. The mean intensity (M.I.) for each nucleus or nucleolus was measured. For analysis of cytoplasmic NF-κB p50 and p65 subunits, the green channel was used; the cytosol of each cell was manually outlined using the freehand selection tool of ImageJ, and the mean intensity (M.I.) was recorded. The results are expressed as the nuclear/cytoplasmic ratios of the p50 and p65 subunits of NF-κB. In addition, the percentage of cells expressing the p50 subunit of NF-κB in the nucleolus was analyzed.

Double immunofluorescence of the microglial marker Iba-1 (1:300; Abcam) or the astroglial marker GFAP (1:400; Sigma-Aldrich) and the p50 subunit of NF-κB were to confirm the expression of NF-κB p50 in these glial cell types.

### Analysis of the role of the TNFR1-NF-κB pathway in the induction of TNF-a synthesis in the Purkinje neurons of hyperammonemic rats

Control and hyperammonemic rats were sacrificed after 1, 2, or 4 weeks of hyperammonemia, and the cerebella were immediately immersed in ice-cold Krebs buffer (in mmol/L: 119 NaCl, 2.5 KCl, 1 KH_2_PO_4_, 26.2 NaHCO_3_, 2.5 CaCl_2_, and 11 glucose) aerated with 95% O_2_ and 5% CO_2_ at pH 7.4. Cerebellar slices (400-μm thick, transverse) were cut and incubated for 20 min at 35.5 °C in Krebs buffer for stabilization. To assess whether the nuclear translocation of NF-κB and the induction of TNF-a synthesis in neurons are mediated by the activation of the TNFR1-TRADD/RIP1/TRAF2 complex, we blocked the formation of this complex by incubation with 20 μM R7050 [38] for 30 min. Then, the slices were fixed in 4% paraformaldehyde in 0.1 M phosphate buffer (pH 7.4) for 24 h at 4 °C. Paraffin-embedded sections (5-μm thick) were cut and mounted on coated glass slides. The sections were then used to analyze TNF-a content in the white matter and Purkinje layer and the subcellular distribution of NF-κB p50 by immunohistochemistry as described above.

### Statistical analysis

The results are expressed as the mean ± standard error. All statistical analyses were performed using GraphPad Prism software. Normality was assessed using the D’Agostino and Pearson Omnibus test and the Shapiro-Wilk normality test. Differences in variances of normally distributed data were assessed using Bartlett’s test. The data were analyzed by a parametric one-way analysis of variance (ANOVA) followed by Tukey’s post hoc test when there were more than two groups, while Student’s *t* test was applied to compare two groups. A confidence level of 95% was accepted as significant. The number of rats used to measure each parameter is indicated in the corresponding figure legend.

## Results

**Fig. 2:**
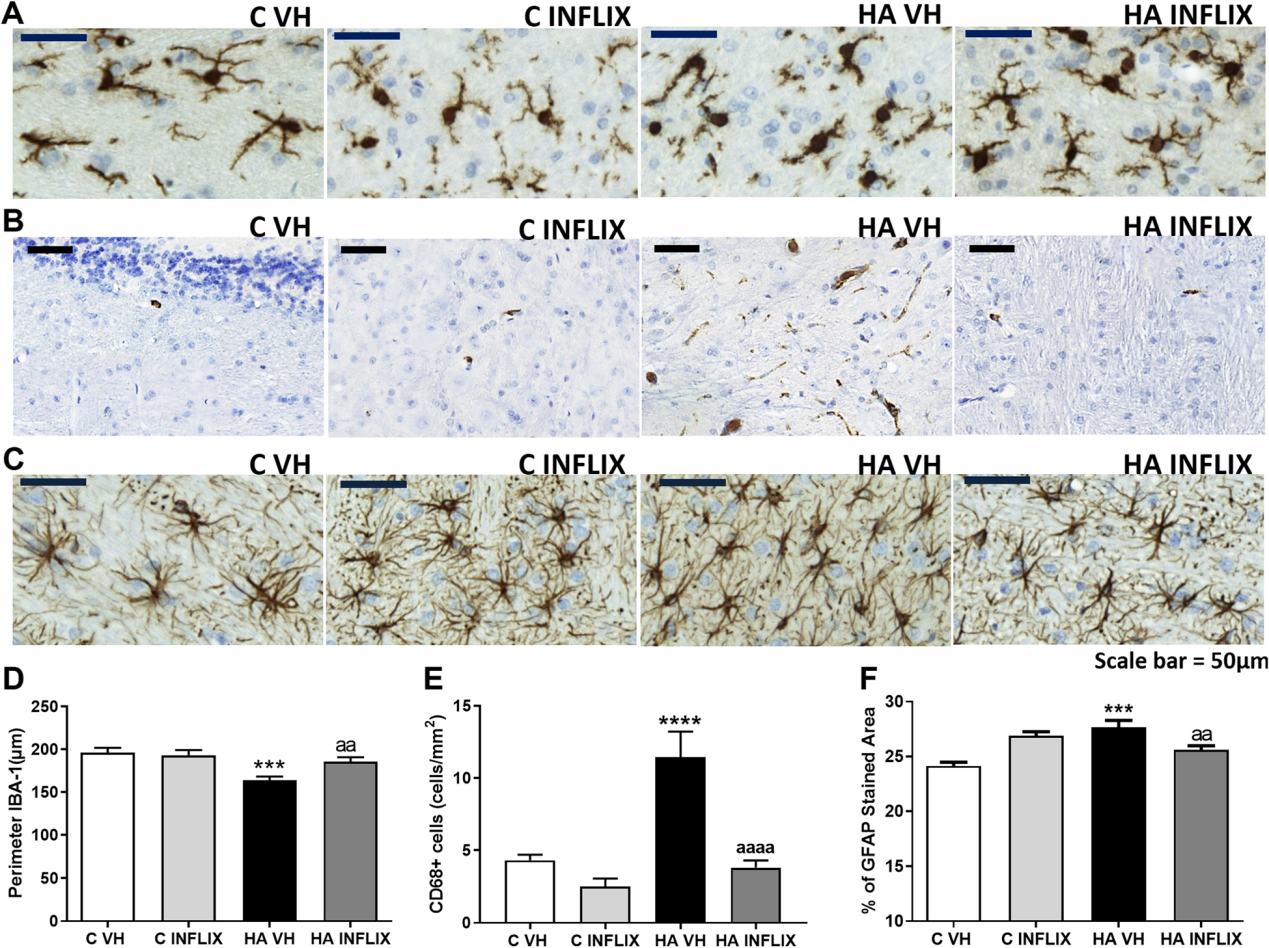
Infliximab prevents microglial and astrocyte activation in the white matter of the cerebellum of hyperammonemic rats. Representative images of microglial (**a**, **b**) and astrocyte activation (**c**) in the white matter of the cerebellum are shown. The perimeter of microglial cells (**d**), cells expressing CD68 (**e**), and the GFAP-stained area (**f**) were evaluated. Hyperammonemia (4 weeks) induced microglial and astrocyte activation in the white matter of the cerebellum, which was prevented by infliximab. The values are the mean ± SEM of 6 rats per group. Values that were significantly different from those of control rats are indicated by asterisks, and values that were significantly different from those of HA rats are indicated by a. ***p < 0.001, ****p < 0.0001, aa *p* < 0.01, aaaa *p* < 0.0001. Scale bar = 50 μm. C VH = vehicle-treated control rats, C INFLIX = control rats treated with infliximab, HA VH = vehicle-treated hyperammonemic rats, HA INFLIX = hyperammonemic rats treated with infliximab

After 4 weeks of hyperammonemia, rats showed activated microglia along with decreases in the length and number of processes and an increase in cell body size (Fig. 2a). These morphological changes were reflected by a reduction (*p* < 0.001) in perimeter (164 ± 1 μm) compared with that observed in control rats (196 ± 6 μm) (Fig. 2d). Treatment with infliximab completely prevented this activation of microglia. The perimeter of microglia in hyperammonemic rats treated with infliximab was 186 ± 5 μm, similar to that of control rats treated with infliximab (193 ± 6 μm) and untreated control rats (Fig. 2d).

Microglial activation was also analyzed by assessing the effects of hyperammonemia on CD68, a specific marker of activated microglia (Fig. 2b). Hyperammonemia strongly increased (*p* < 0.0001) the number of cells stained with CD68 in the white matter to 11 ± 2 cells/mm^2^ compared to 4 ± 1 cells/mm^2^ in control rats (Fig. 2e). Treatment with infliximab prevented the activation of microglia, reducing the number of cells expressing CD68 to 4 ± 1 cells/mm^2^, which was similar to that in control rats (Fig. 2e).

Hyperammonemia also induced the activation of astrocytes in the white matter of the cerebellum, which was also prevented by treatment with infliximab (Fig. 2c). The GFAP-stained area was increased (*p* < 0.001) in hyperammonemic rats (28 ± 1%) compared to control rats (24 ± 1%) (Fig. 2f). Peripheral treatment with infliximab also reduced astrocyte activation in hyperammonemic rats; there was less GFAP staining (26 ± 1%) in the white matter of these rats than in the white matter of untreated rats (Fig. 2f).

**Fig. 3:**
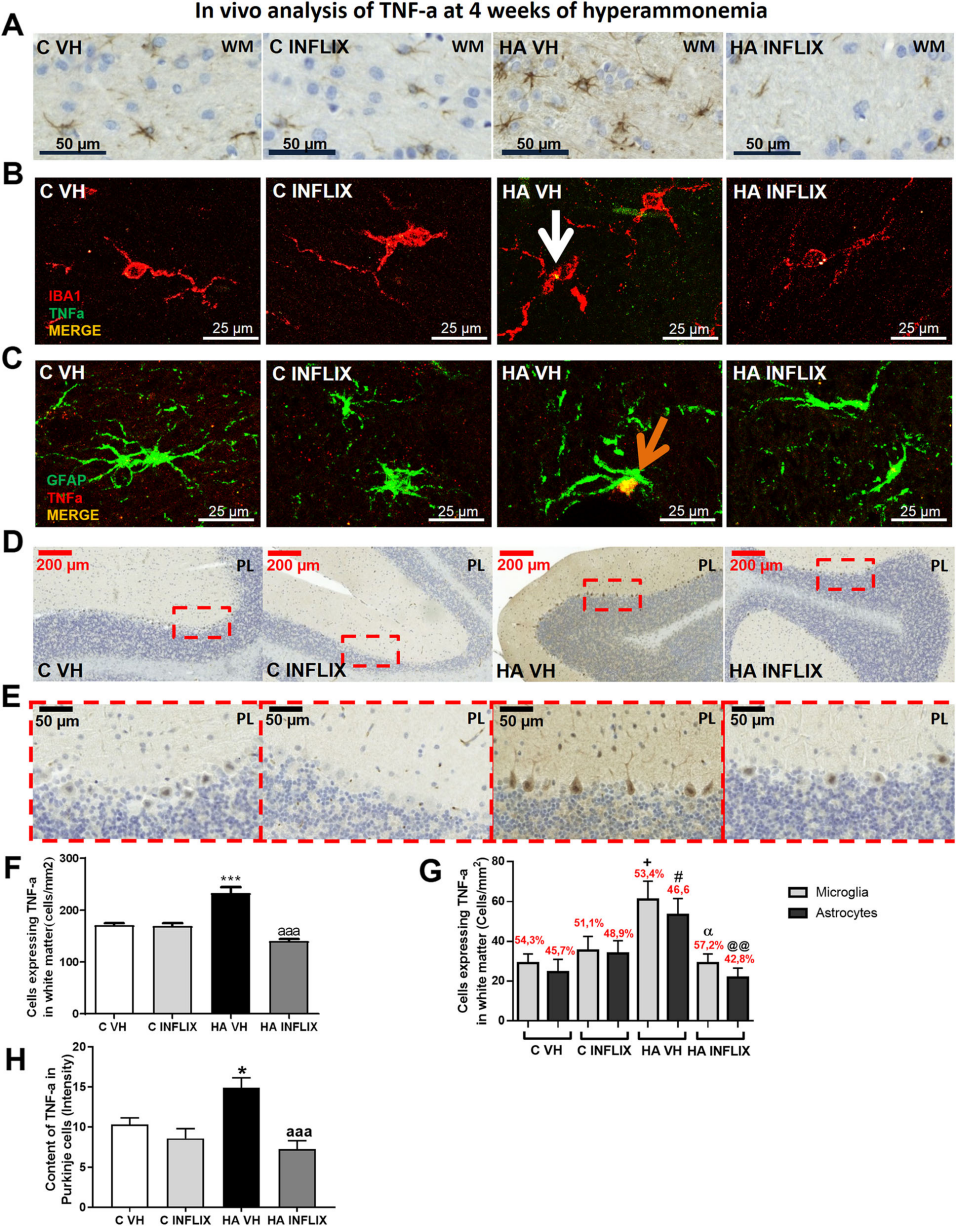
The content of TNF-a is increased in microglia, astrocytes, and Purkinje neurons after 4 weeks of hyperammonemia and is normalized by infliximab. Immunohistochemistry for TNF-a was performed as described in the “Methods” section. Representative images of white matter (**a**) and Purkinje neurons (**d**, **e**) are shown, and the quantified data are shown in **f** and **h**. Double immunofluorescence was performed using antibodies against TNF-a and Iba-1 (**b**) or GFAP (**c**), and the percentage of glial cells expressing TNF-a was determined (**g**). Values that were significantly different from those of control rats are indicated by asterisks, and values that were significantly different from those of HA VH rats are indicated by a. + refers to microglia expressing TNF-a in HA VH rats vs microglia expressing TNF-a in C VH rats, # refers to astrocytes expressing TNF-a in HA VH rats vs astrocytes expressing TNF-a in C VH rats, *α* refers to microglia expressing TNF-a in HA VH rats vs microglia expressing TNF-a in HA INFLIX rats, and @ refers to astrocytes expressing TNF-a in HA VH rats vs astrocytes expressing TNF-a in HA INFLIX rats. One symbol indicates *p* ≤ 0.05; two symbols indicate *p* < 0.01 and three symbols indicate *p* ≤ 0.001. C VH = vehicle-treated control rats; C INFLIX = control rats treated with infliximab; HA VH = vehicle-treated hyperammonemic rats; HA INFLIX = hyperammonemic rats treated with infliximab

We then analyzed the effects of hyperammonemia and infliximab treatment on the expression of TNF-a in the white matter by immunohistochemistry (Fig. 3). The number of cells expressing TNF-a was increased (*p* < 0.001) to 233 ± 12 mm^2^ in hyperammonemic rats compared to 171 ± 4 mm^2^ in control rats (Fig. 3a). Treatment with infliximab completely prevented this increase (141 ± 4 mm^2^) (Fig. 3a, f). To assess whether this increase occurs in microglia or astrocytes, we performed double fluorescence staining for TNF-a and Iba-1 (Fig. 3b) or GFAP (Fig. 3c). Increases in TNF-a in the white matter were observed both in microglia (Fig. 3b, white arrow; *p* < 0.05) and astrocytes (Fig. 3c, orange arrow; *p* < 0.05) to a similar extent (Fig. 3g).

Unexpectedly, we also observed an increase in TNF-a expression in Purkinje neurons in rats after 4 weeks of hyperammonemia (Fig. 3d, e, and h). The intensity of TNF-a staining in Purkinje neurons was higher in hyperammonemic rats (15 ± 1) than in control rats (10 ± 1) (Fig. 1h). Treatment with infliximab prevented the increase in TNF-a in Purkinje neurons (7 ± 1) (Fig. 3h).

**Fig. 4:**
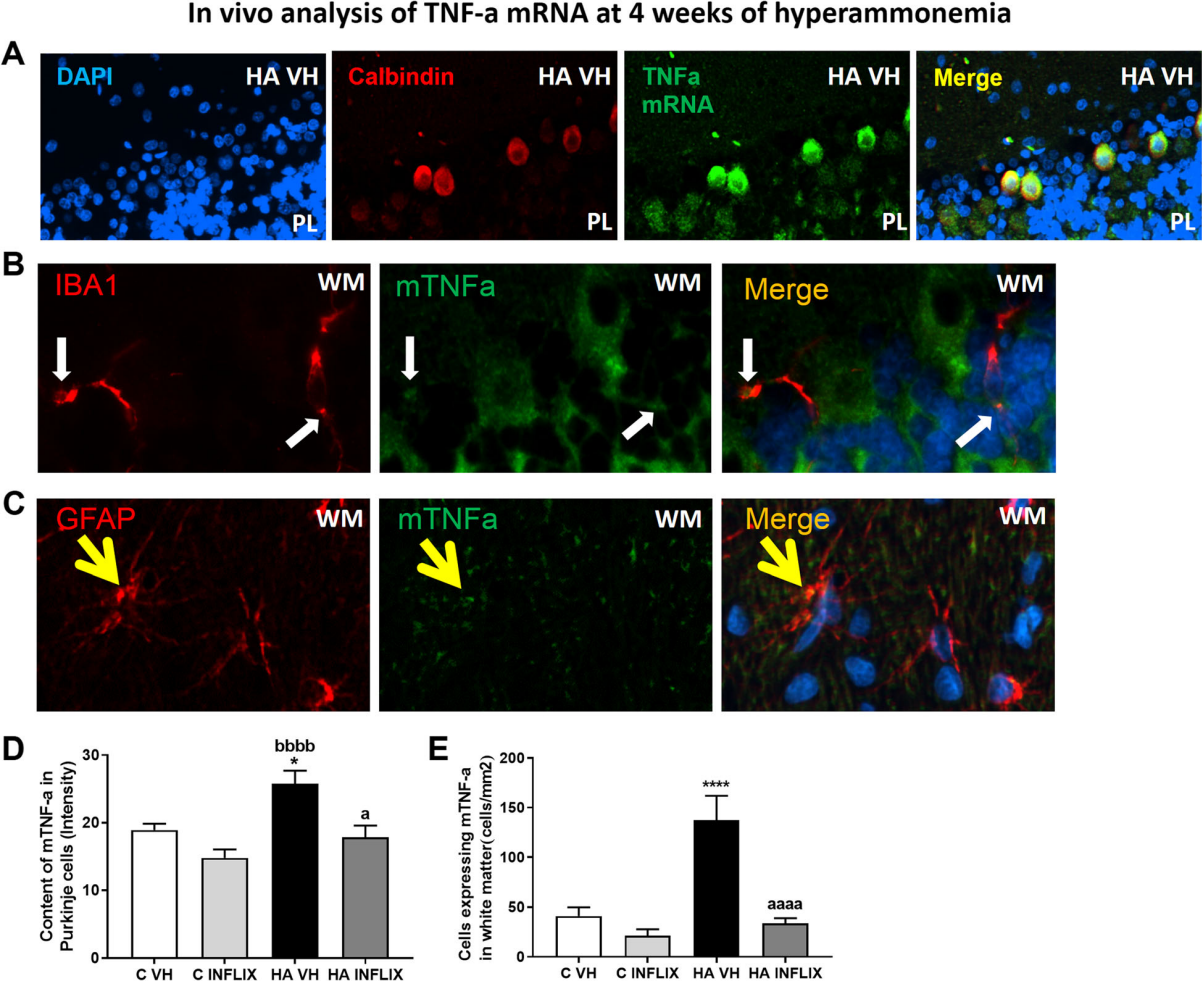
The expression of mTNF-a is increased in the white matter and Purkinje layer of the cerebellum after 4 weeks of hyperammonemia and is normalized by infliximab. Double fluorescence staining for TNF-a mRNA (green) and calbindin (red, **a**), Iba-1 (red, **b**), or GFAP (red, **c**) is shown. The content of TNF-a mRNA in Purkinje neurons (**d**) and white matter (**e**) was quantified. The values are the mean ± SEM of 3 rats per group. Values that were significantly different from those of the control rats are indicated by asterisks, values that were significantly different from those of the HA VH rats are indicated by a, and values that were significantly different from those infliximab-treated control rats are indicated by b. *p ≤ 0.05; ****p ≤ 0.0001; a *p* < 0.05; aaaa *p* ≤ 0.0001; bbbb *p* ≤ 0.0001. C VH = vehicle-treated control rats; C INFLIX = control rats treated with infliximab; HA VH = vehicle-treated hyperammonemic rats; HA INFLIX = hyperammonemic rats treated with infliximab

To confirm that staining for TNF-a in Purkinje neurons is due to its synthesis in these neurons, we analyzed the content of TNF-a mRNA by fluorescence in situ hybridization (Fig. 4a, d). Compared to control rats (19 ± 1) and hyperammonemic rats treated with infliximab (18 ± 2), hyperammonemic rats showed a higher level of TNF-a mRNA (mean intensity 26 ± 2) in Purkinje neurons (Fig. 4a, d).

The mRNA expression of TNF-a was also increased in glial cells in the white matter of hyperammonemic rats (137 ± 24 cells/mm^2^; *p* < 0.0001) compared to control rats (41 ± 9 cells/mm^2^, Fig. 4e). Hyperammonemic rats treated with infliximab showed values similar to control rats (34 ± 5 cells/mm^2^) (Fig. 4e). Again, double immunofluorescence showed that increases in TNF-a mRNA in the white matter occurred both in microglia (Fig. 4b, white arrow) and in astrocytes (Fig. 4c, yellow arrow).

**Fig. 5:**
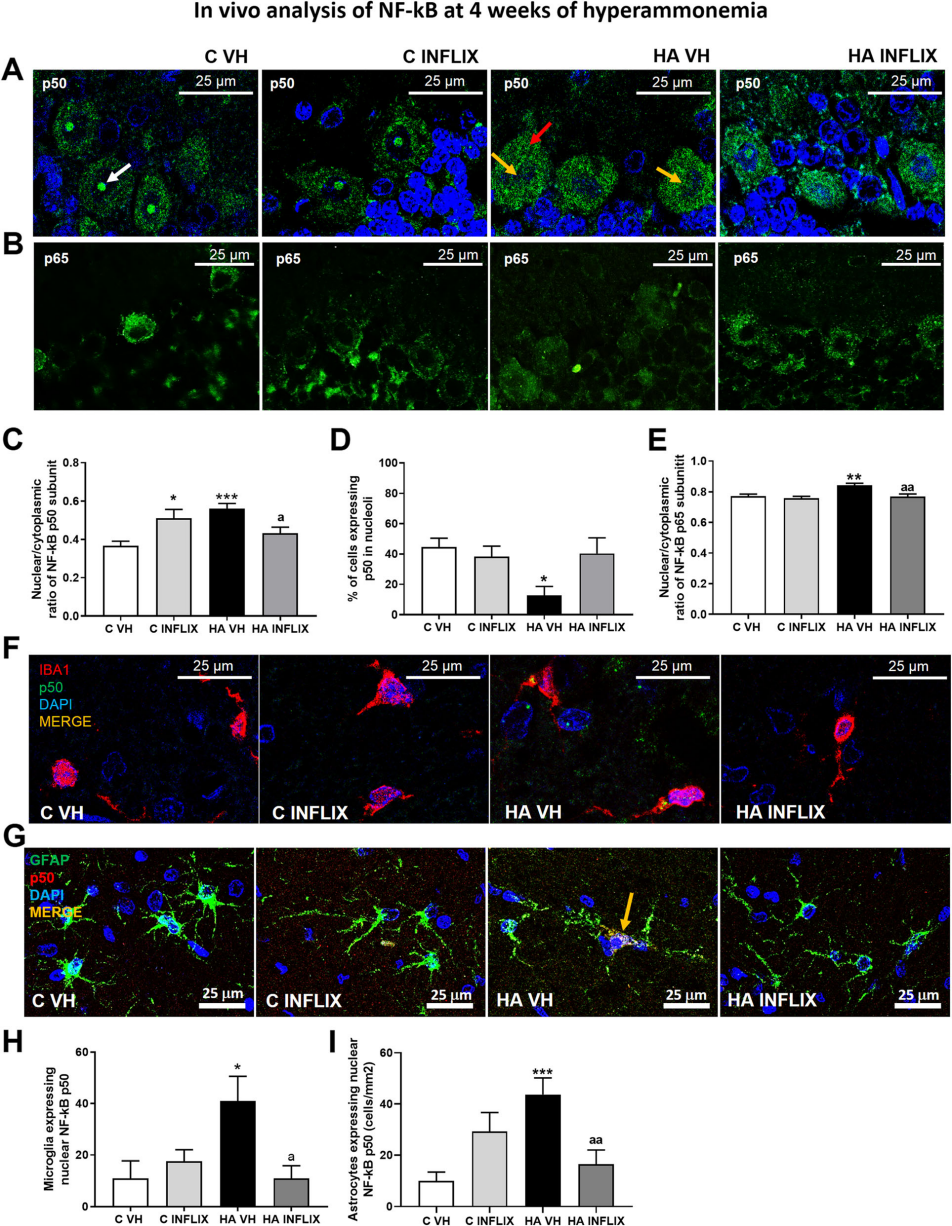
Sustained hyperammonemia increases the nuclear translocation of the p50 and p65 subunits of NF-KB in Purkinje neurons and glial cells, which is prevented by infliximab. Analysis of NF-kB in neurons was performed by immunofluorescence using antibodies against the p50 (**a**) and p65 (**b**) subunits of NF-kB (green staining). Nuclei were stained blue with DAPI. The nuclear/cytoplasmic ratios of p50 (**c**) and p65 (**e**) and the proportion of cells containing p50 in the nucleoli (**d**) are quantified. To analyze the nuclear translocation of NF-kB in glial cells, double immunofluorescence was performed for NF-kB p50 (green in **f** and red in **g**) and Iba-1, a microglial marker (red in F), or GFAP, an astroglial marker (green in **g**). The merged images show the colocalization of these proteins (yellow). The number of microglia (H) and astrocytes (**i**) expressing nuclear p50 was quantified. The values are the mean ± SEM of 3–4 rats per group. Values that were significantly different from those of the control rats are indicated by asterisks, and values that were significantly different from those of the HA VH rats are indicated by a. *p ≤ 0.05, **p ≤ 0.01, ***p < 0.001, a *p* < 0.05; aa *p* ≤ 0.01. C VH = vehicle-treated control rats, C INFLIX = control rats treated with infliximab, HA VH = vehicle-treated hyperammonemic rats, HA INFLIX = hyperammonemic rats treated with infliximab

We then assessed whether hyperammonemia can induce TNF-a expression in Purkinje neurons through the p50 and p65 subunits of NF-κB (Fig. 5). Hyperammonemia strongly altered the intracellular distribution of p50, increasing the nuclear/cytosolic ratio (0.56 ± 0.03) compared to that in control rats (0.37 ± 0.02) (Fig. 5a, c) and strongly reducing the level of p50 in the nucleolus (Fig. 5a, d). Treatment with infliximab prevented all these changes in the distribution of p50 (Fig. 5c, d).

The intensity of staining for the p65 subunit of NF-κB in the nucleus was also increased in the Purkinje neurons of hyperammonemic rats, and this was also prevented by infliximab (Fig. 5b, e).

The expression of p50 of NF-κB was also increased in glial cells in hyperammonemic rats (Fig. 5f–i). The number of cells expressing p50 in microglia and astrocytes was increased more than 3-fold in hyperammonemic rats, and this was prevented by treatment with infliximab (Fig. 5f–i).

**Fig. 6:**
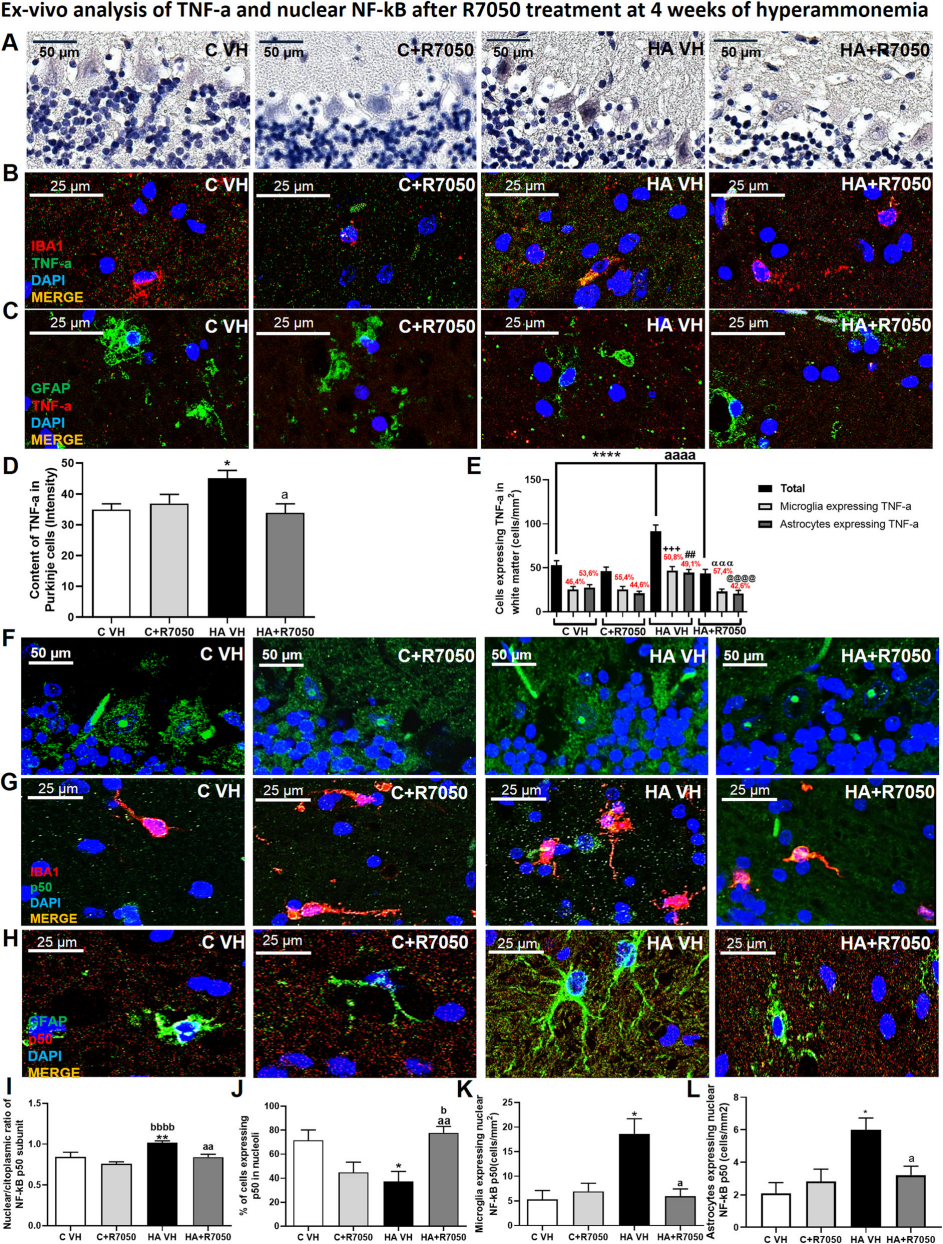
TNF-a synthesis in the cerebellum is TRADD/RIP1/TRAF2 complex formation-dependent. Analysis of TNF-a expression and NF-kB p50 subunit nuclear translocation was performed in slices incubated with R7050 as described in the “Material and methods” section. The expression of TNF-a was reduced in neurons (**a**, **d**), microglia (Iba-1: red; TNF-a: green; **b**, **e**) and astrocytes (GFAP: green; TNF-a: red; **c**, **e**) in hyperammonemic rats after 30 min of incubation with R7050. These effects were due to a reduction in the nuclear NF-kB p50 in Purkinje neurons (**f**, **i**), microglia (**g**, **k**), and astrocytes (**h**, **l**). R7050 treatment also restored the nucleolar translocation of the p50 subunit in Purkinje neurons (**j**). The values are the mean ± SEM of 4 rats per group. Values that were significantly different from those of the control rats are indicated by asterisks, and values that were significantly different from those of the HA VH rats are indicated by a. + refers to microglia expressing TNF-a in HA VH rats vs microglia expressing TNF-a in C VH rats, # refers to astrocytes expressing TNF-a in HA VH rats vs microglia expressing TNF-a in HA + R7050 rats, and @ refers to astrocytes expressing TNF-a in HA VH rats vs astrocytes expressing TNF-a in HA + R7050 rats. One symbol indicates *p* ≤ 0.05, two symbols indicate *p* < 0.01, three symbols indicate *p* ≤ 0.001, and four symbols indicate *p* < 0.0001. C VH = cerebellar slices obtained from vehicle-treated control rats, C + R7050 = cerebellar slices obtained from control rats and incubated with R7050, HA VH = cerebellar slices obtained from vehicle-treated hyperammonemic rats, HA + R7050 = cerebellar slices obtained from hyperammonemic rats and incubated with R7050

We then assessed whether the induction of NF-κB p50 nuclear translocation and TNF-a expression in hyperammonemic rats is mediated by the activation of the TNF-a receptor TNFR1. To do this, we blocked the formation of the TNFR1-TRADD/RIP1/TRAF2 complex using R7050. These experiments were performed ex vivo in cerebellar slices freshly isolated from control and hyperammonemic rats. Incubation with R7050 reduced TNF-a expression in the Purkinje neurons (*p* < 0.05; Fig. 6a), microglia (*p* < 0.0001; Fig. 6b) and astrocytes (*p* < 0.0001; Fig. 6c) of hyperammonemic rats to values similar to those of control rats (Fig. 6d, e).

We then assessed whether the normalization of TNF-a levels by R7050 is associated with the normalization of the subcellular distribution of NF-κB p50. R7050 normalized the nuclear/cytosolic distribution of p50 and restored the nucleolar content of p50 in the Purkinje neurons of hyperammonemic rats (Fig. 6f, i, and j). R7050 also normalized the expression of p50 in microglia (Fig. 6g, k) and astrocytes (Fig. 6h, l) in the white matter of hyperammonemic rats.

All the above studies were performed after the rats were exposed to hyperammonemia for 4 weeks and showed that hyperammonemia induced TNF-a expression in Purkinje neurons. As this is not a common situation, we assessed whether sustained hyperammonemia is necessary for this induction of TNF-a and whether such induction also occurs after shorter periods of hyperammonemia.

**Fig. 7:**
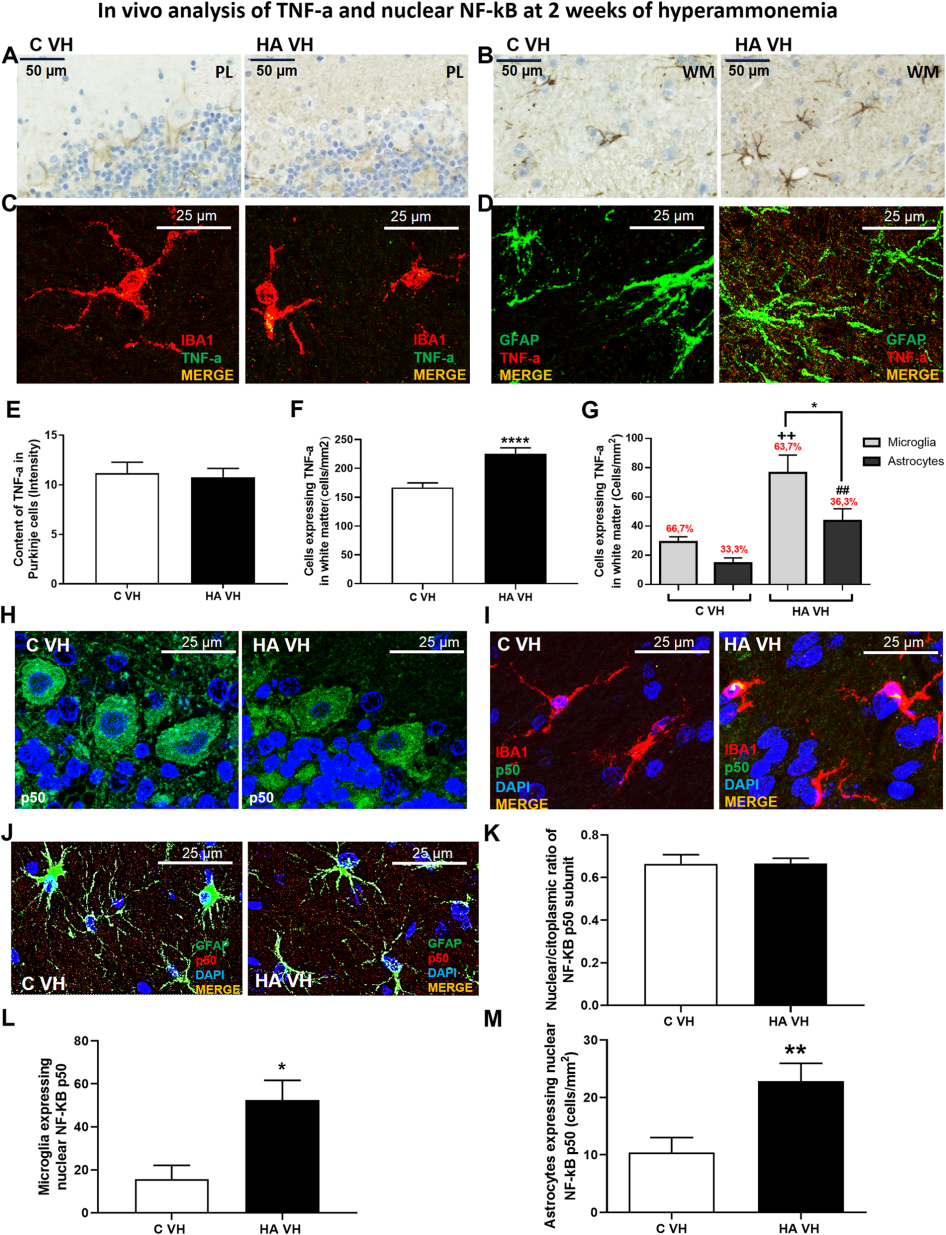
TNF-a is increased in glial cells but not in Purkinje neurons after 2 weeks of hyperammonemia. An increase in TNF-a was observed in glial cells (white matter, **b**, **e**) but not in Purkinje neurons (**a**, **d**). Double immunofluorescence confirmed the presence of TNF-a in microglia (**c**) and astrocytes (**d**), and the proportion of these cells expressing TNF-a was quantified (**f**). To investigate the contribution of the nuclear translocation of NF-kB to TNF-a synthesis, immunofluorescence for the p50 subunit was performed in the Purkinje layer, and representative images are shown in **g** (green staining) and quantified in **j**. Double immunofluorescence was also performed for NF-kB p50 (green in **h** and red in **i**) and Iba-1, a microglial marker (red in **h**) or GFAP, an astroglial marker (green in **i**). The merged image shows the colocalization of these proteins (yellow). The number of microglia and astrocytes expressing nuclear p50 are quantified in **k** and **l**, respectively. The values are the mean ± SEM of 3 rats per group. Values that were significantly different from those of the control rats are indicated by asterisks. *p ≤ 0.05, **p ≤ 0.051, ****p ≤ 0.0001. ++ refers to microglia expressing TNF-a in HA VH rats vs microglia expressing TNF-a in C VH rats (*p* < 0.01) and ## refers to astrocytes expressing TNF-a in HA VH rats vs astrocytes expressing TNF-a in C VH rats (*p* < 0.01). C VH = vehicle-treated control rats, HA VH = vehicle-treated hyperammonemic rats

We repeated the analysis of TNF-a expression after 2 weeks of hyperammonemia (Fig. 7). We did not observe any increase in TNF-a in Purkinje neurons (Fig. 7a, e), while TNF-a was increased in glial cells in the white matter (*p* < 0.01; Fig. 7b, f), both in microglia (Fig. 7c, g) and astrocytes (Fig. 7d, g). The increase in TNF-a was slightly higher in microglial cells than in astrocytes (Fig. 7g).

We also analyzed the nuclear translocation of NF-κB p50 after 2 weeks of hyperammonemia (Fig. 7h–m). In agreement with its lack of an effect on TNF-a, we did not observe any effect of hyperammonemia on the subcellular distribution of p50 in Purkinje neurons, with the distribution remaining similar to that observed in control rats (Fig. 7h, k). Additionally, in agreement with the increase in TNF-a, rats exposed to 2 weeks of hyperammonemia showed increased expression of p50 in microglia (Fig. 7i, l) and astrocytes (Fig. 7j, m).

**Fig. 8:**
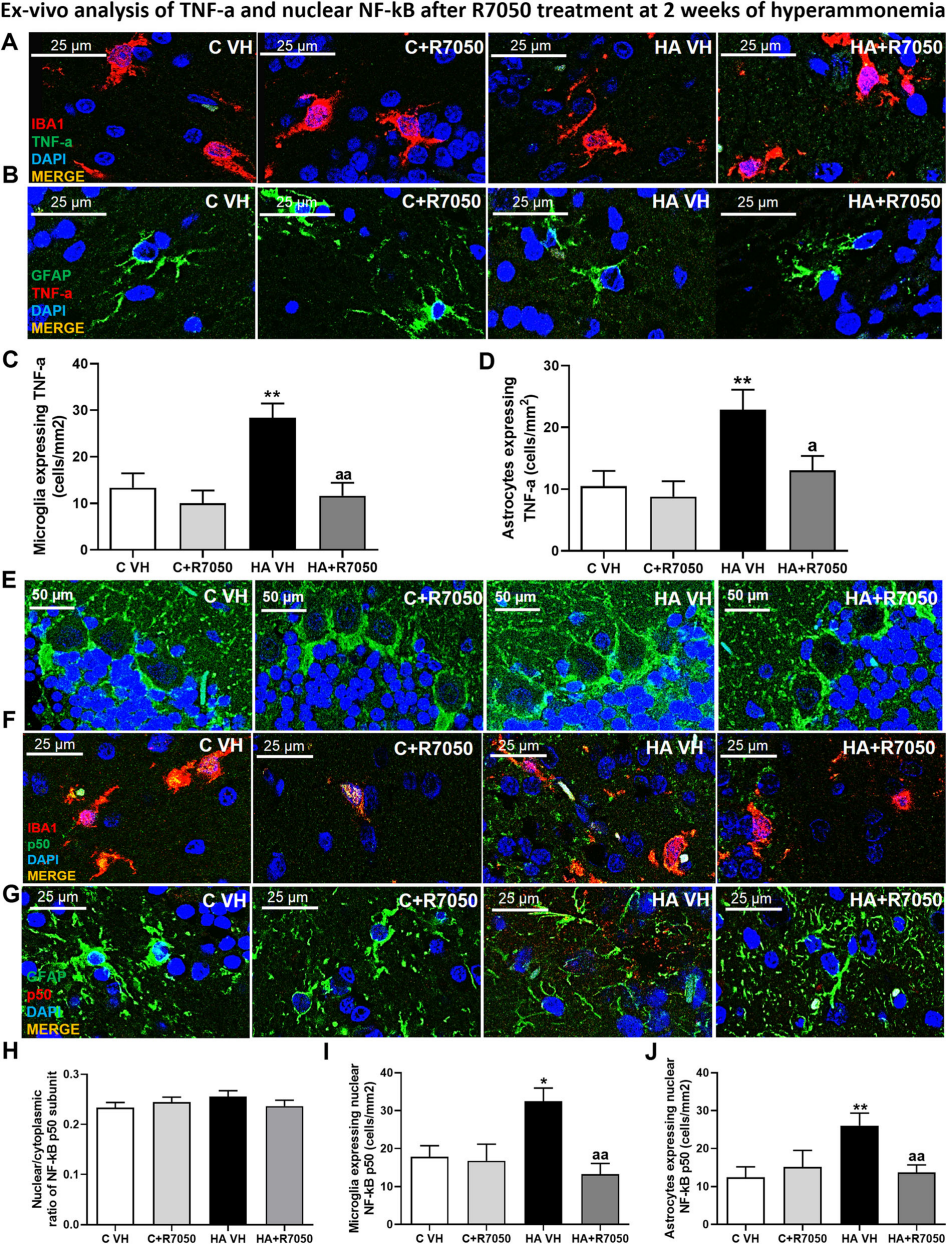
Analysis of the TNF-a-TNFR1-NF-κB pathway after 2 weeks of hyperammonemia. The effects of R7050 treatment on TNF-a expression in microglia (**a**, **c**) and astrocytes (**b**, **d**) and on NF-kB p50 nuclear translocation in Purkinje neurons (**e**, **h**), microglia (**f**, **i**) and astrocytes (**g**, **j**) after 2 weeks of hyperammonemia are shown. The values are the mean ± SEM of 3 rats per group. Values that were significantly different from those of the control rats are indicated by asterisks, and values that were significantly different from those of HA VH rats are indicated by a. *p ≤ 0.05, **p ≤ 0.01, a < 0.05, and aa *p* ≤ 0.01. C VH = cerebellar slices obtained from vehicle-treated control rats; C + R7050 = cerebellar slices obtained from control rats and incubated with R7050; HA VH = cerebellar slices obtained from vehicle-treated hyperammonemic rats, HA + R7050 = cerebellar slices obtained from hyperammonemic rats and incubated with R7050

We also used R7050 to assess the role of TNFR1 activation in the induction of NF-κB p50 nuclear translocation and TNF-a expression after 2 weeks of hyperammonemia (Fig. 8).

Incubation with R7050 reduced TNF-a expression in the microglia (*p* < 0.001; Fig. 8a, c) and astrocytes (*p* < 0.005; Fig. 8b, d) of hyperammonemic rats to values similar to those of control rats (Fig. 8c, d).

After 2 weeks of hyperammonemia, the nuclear/cytosolic distribution of p50 was not altered in Purkinje neurons or by R7050 (Fig. 8e, h). R7050 normalized the expression of p50 in microglia (Fig. 8f, i) and astrocytes (Fig. 8g, j) in the white matter of rats exposed to 2 weeks of hyperammonemia.

**Fig. 9:**
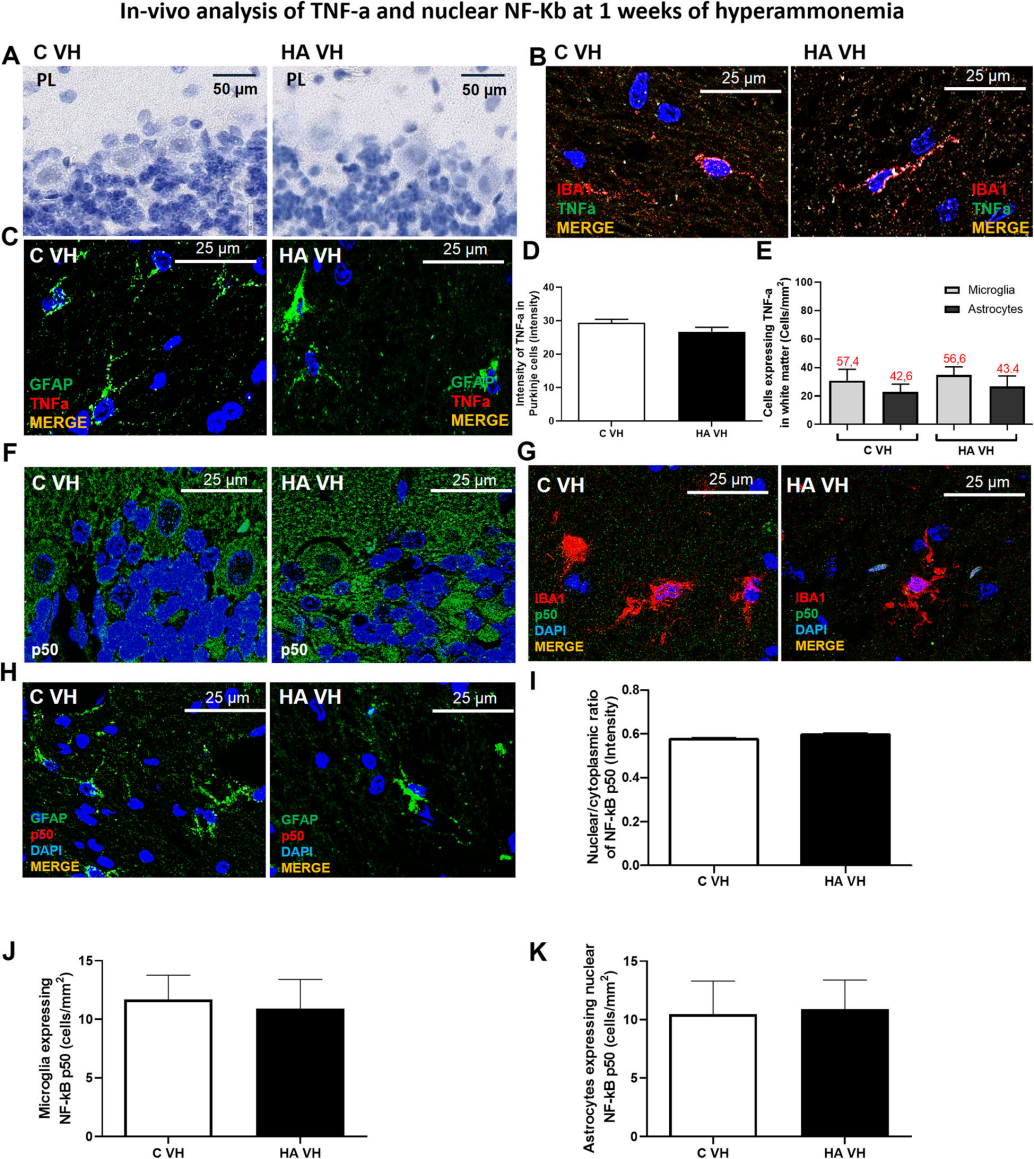
TNF-a and nuclear NF-kB are not increased in hyperammonemic rats at 1 week. Representative images of TNF-a expression and nuclear NF-kB in Purkinje neurons (**a**, **f**, respectively), microglia (**b**, **g**, respectively), and astrocytes (**c**, **h**, respectively) are shown. The content of TNF-a and the nuclear/cytoplasmic ratio of the p50 subunit of NF-kB are quantified in **d** and **i**, respectively. **e** The percentage of microglia vs astrocytes expressing TNF-a. The numbers of microglia and astrocytes expressing nuclear p50 are quantified in **j** and **k**, respectively. C VH = vehicle-treated control rats, HA VH = vehicle-treated hyperammonemic rats

**Fig. 10:**
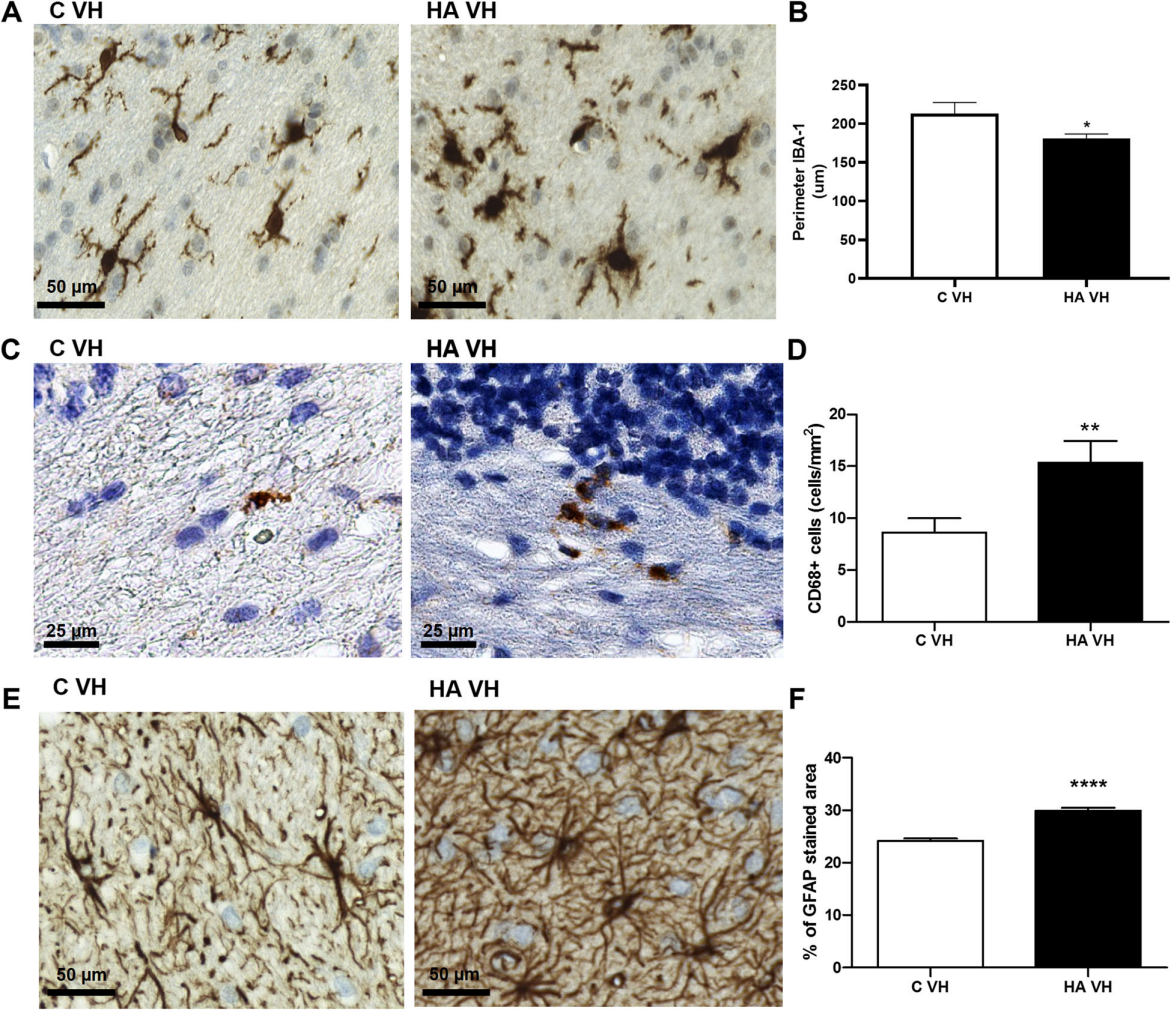
Microglia and astrocytes are already activated after only 1 week of hyperammonemia. Representative images of microglial (**a**, **c**) and astrocyte activation (**e**) in the white matter of the cerebellum are shown. The perimeter of microglial cells (**b**), cells expressing CD68 (**d**), and the GFAP-stained area (**f**) was evaluated. The values are the mean ± SEM of 3 rats per group. Values that were significantly different from those of the control rats are indicated by asterisks. *p < 0.05, **p < 0.01, and ****p < 0.0001. C VH = vehicle-treated control rats, HA VH = vehicle-treated hyperammonemic vehicle rats

We then analyzed the changes in TNF-a expression and NF-κB after 1 week of hyperammonemia (Fig. 9). At this time, we did not observe any increase in TNF-a expression in Purkinje neurons (Fig. 9a, d), microglia (Fig. 9b, e), or astrocytes (Fig. 9c, e). NF-κB expression also remained unaltered in Purkinje neurons (Fig. 9f, i), microglia (Fig. 9g, j) and astrocytes (Fig. 9h, k). Despite this lack of an effect on TNF-a and NF-κB after 1 week of hyperammonemia, both microglia and astrocytes were activated in the cerebellum. This was reflected by the reduced perimeter of microglia (Fig. 10a, b), increased expression of CD68 (Fig. 10c, d), and increased GFAP-stained area (Fig. 10e, f).

**Fig. 11:**
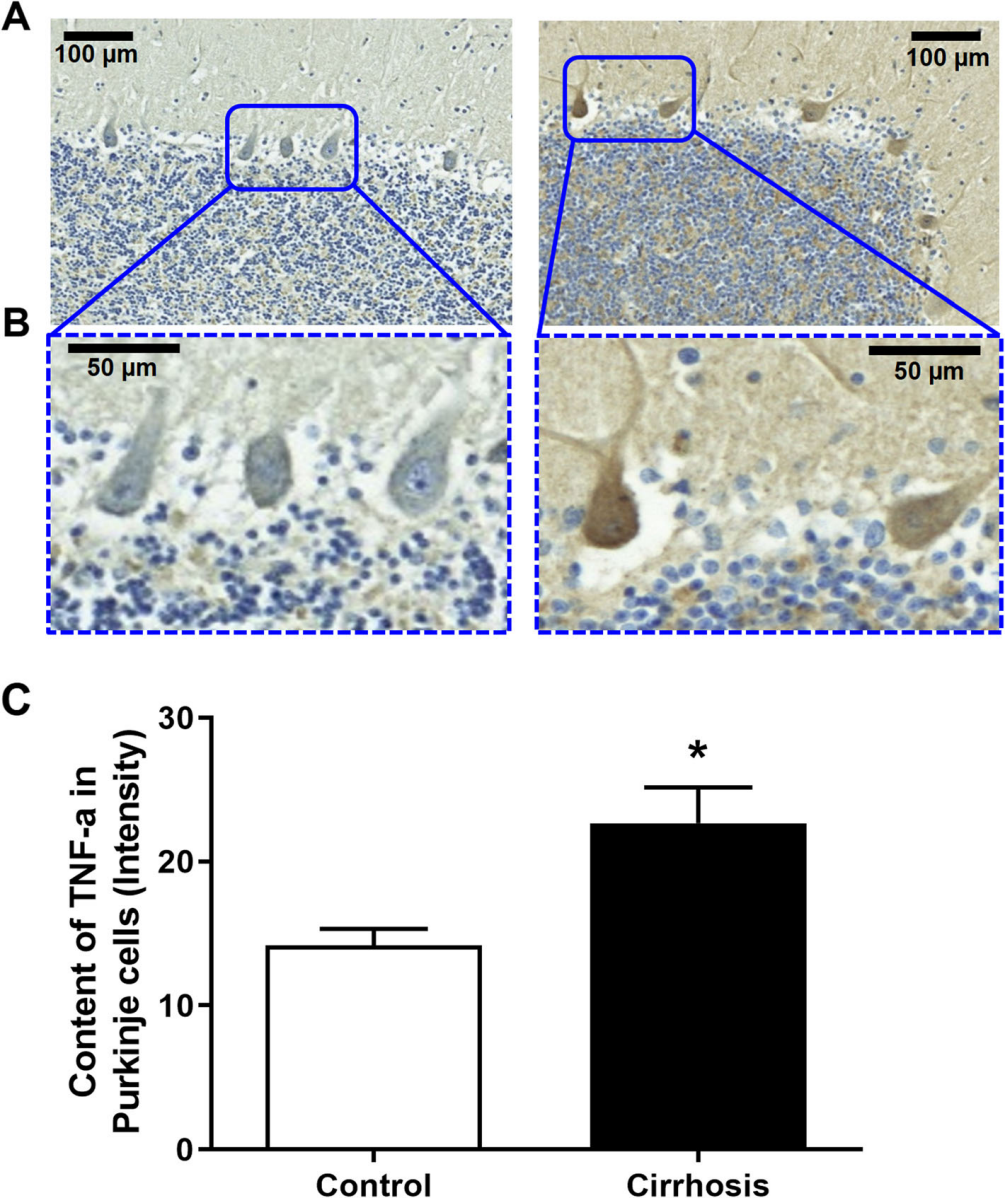
TNF-a is increased in the Purkinje neurons of patients who die with liver cirrhosis. Representative images of Purkinje cells expressing TNF-a are shown in **a** and **b** and quantified in **c**. The values are the mean ± SEM of 3–4 subjects per group. Values that were significantly different from those of the control rats are indicated by asterisks. *p < 0.05

Patients with liver cirrhosis show sustained hyperammonemia. We assessed whether this also leads to an increased level of TNF-a in Purkinje neurons. As shown in Fig. 11, this was the case. The Purkinje neurons of patients who died with liver cirrhosis exhibited increased TNF-a content (*p* = 0.01) compared to that in control subjects who died without liver or neurodegenerative diseases.

## Discussion

This study shows that sustained (4 weeks), but not short-term (2 weeks), hyperammonemia induces the expression of TNF-a in Purkinje neurons in rats. Moreover, TNF-a expression is also increased in Purkinje neurons of patients who die with liver cirrhosis and are exposed to sustained hyperammonemia. As discussed below, this induction of TNF-a may contribute to the neurological alterations observed in hyperammonemia and hepatic encephalopathy by altering the function of Purkinje neurons and neurotransmission and by inducing neuronal death.

**Fig. 12:**
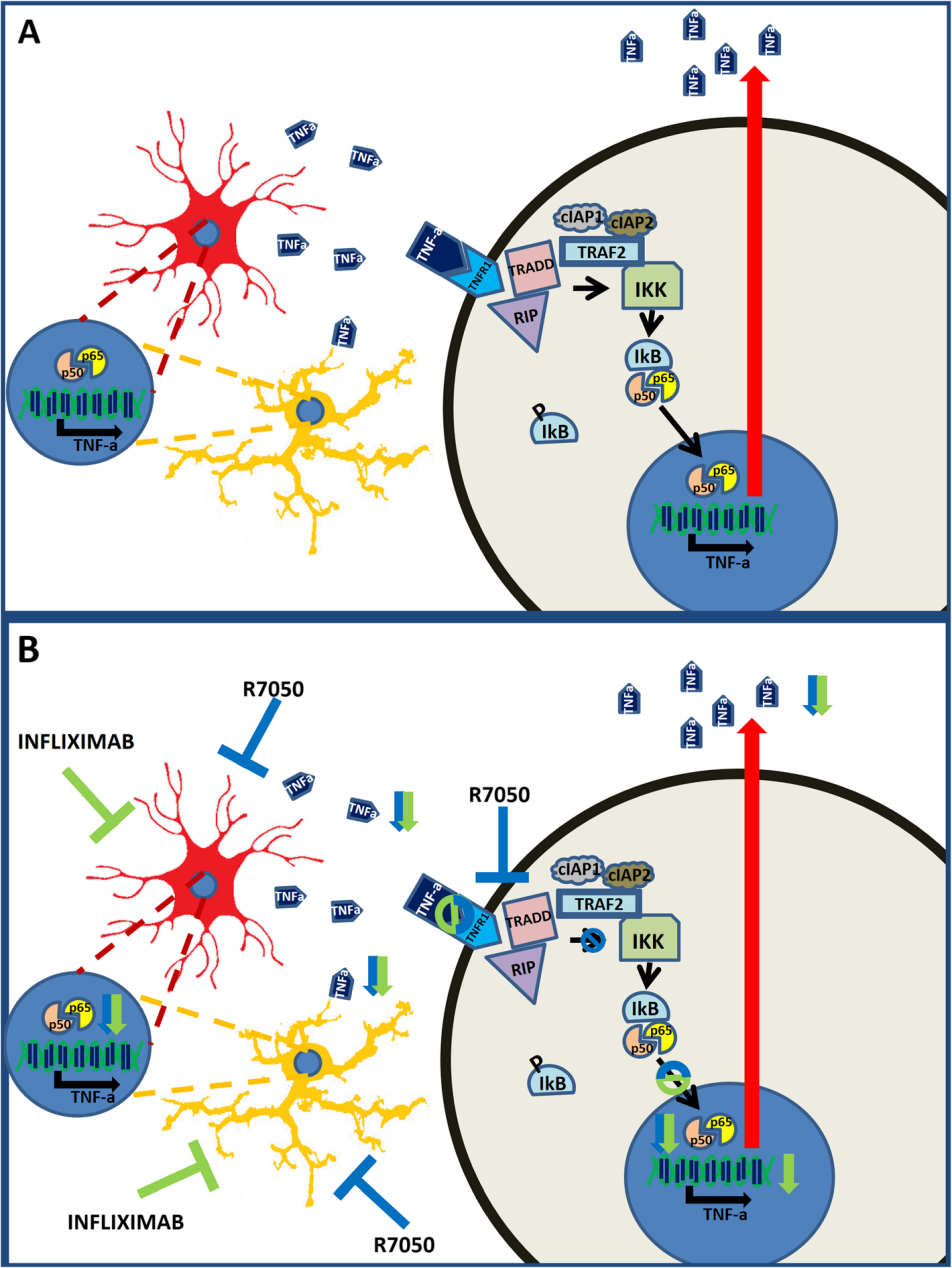
Proposed mechanisms by which chronic hyperammonemia increases TNF-a in neurons and glial cells. **a** Chronic hyperammonemia induces the activation of microglia and astrocytes in the white matter of the cerebellum. Activated glial cells increase the nuclear translocation of NF-kB, which induces the transcription of pro-inflammatory TNF-a. Glial TNF-a binds to the TNFR1 receptor in Purkinje cells, leading to the activation of the NF-kB pathway and TNF-a synthesis in these neurons. **b** Peripheral treatment with infliximab reduces glial cell activation and TNF-a synthesis, which prevents the activation of the NF-KB pathway and TNF-a synthesis in Purkinje neurons. Finally, ex vivo treatment of cerebellar slices from hyperammonemic rats with R7050 shows that TNF-a synthesis in glial cells and neurons is mediated by the activation of the TNFR1-TRAD/RIP1-NF-κB pathway

The induction of TNF-a in Purkinje neurons is associated with increased nuclear localization of NF-κB, a transcription factor that promotes the transcription of TNF-a mRNA [31]. We show that TNF-a mRNA is also increased in Purkinje neurons, indicating that TNF-a is synthesized inside Purkinje cells, likely due to the increased nuclear translocation of NF-κB (Fig. 12a).

Both the nuclear translocation of NF-κB and the increased level of TNF-a in Purkinje neurons are reversed by blocking the TNFR1-TRADD/RIP1/TRAF2 signal transduction pathway through the activation of the TNF-a receptor TNFR1 (Fig. 12b). This suggests that TNF-a produced in glia activates TNFR1 in Purkinje neurons, inducing the translocation of NF-κB to the nucleus and increasing the transcription of TNF-a mRNA and the synthesis of the TNF-a protein (Fig. 12). Once produced and if not counteracted, TNF-a can cause an autocrine loop both in glia and neurons, further exacerbating the effects of neuroinflammation in the affected brain.

In fact, the data reported here show that hyperammonemia induces the synthesis of TNF-a in activated microglia and astrocytes after 2 weeks of hyperammonemia, when TNF-a was not increased in Purkinje cells. We also show that after 1 week of hyperammonemia, both microglia and astrocytes are activated, but TNF-a expression is not yet induced. This indicates that the process follows a time course in which hyperammonemia induces the activation of microglia and astrocytes at 1 week. This is followed by the induction of TNF-a in both types of glial cells (but not in Purkinje cells) at 2 weeks. TNF-a released by glial cells activates TNFR1 in Purkinje neurons, leading to the induction of TNF-a expression. It is entirely plausible that activated microglia or astrocytes produce a plethora of signals that can cause an increase in TNFa expression in neurons and that treatment with R7050 may act on glia to prevent the release of a number of factors in a TNFR1-dependent manner that may then act on neurons. Further in vitro studies and/or the use of cell-specific TNFR1 knockout models will help to better elucidate the exact mechanisms involved.

We have recently shown using the same animal model used here that hyperammonemia per se induces peripheral inflammation by increasing proinflammatory TNF-a, IL-6, and PGE2 and decreasing anti-inflammatory IL-10 [22]. These factors contribute to neuroinflammation in the hippocampus and to cognitive impairment in rats [22].

Here, we show that blocking peripheral TNF-a with an anti-TNF-a antibody (infliximab) prevents the activation of microglia and astrocytes and the induction of TNF-a, indicating that peripheral TNF-a and inflammation trigger glial activation and all the above process, leading to TNF-a expression in Purkinje neurons. However, the mechanisms by which peripheral inflammatory signals are able to induce glial cell activation and TNF-a expression in the cerebellum are unclear. There are several mechanisms by which systemic inflammation may induce neuroinflammation, including (a) the active transport of certain cytokines into the brain [43]; (b) the activation of blood cytokine receptors by their ligands in endothelial cells, which triggers the release of inflammatory factors into the brain [44]; (c) the direct entry of cytokines into the circumventricular regions due to the lack of an intact blood-brain barrier in these brain areas; and (d) the infiltration of peripheral monocytes or lymphocytes in the brain [45].

TNF-a synthesis in neurons has already been reported in some pathological situations, including spinal cord injury [29], stroke [27], and sciatic nerve injury [30]. TNF-a and IL-1b are expressed in hippocampal neurons in vivo in response to lesions [46] and pneumococcal meningitis [47]. In situ hybridization studies have shown that in murine pneumococcal meningitis, TNF-a mRNA is first upregulated in astroglial cells but is strongly increased in hippocampal neurons at 18–24 h [47]. TNF-a mRNA and protein expression are also induced in neurons in the hippocampus of rats with hepatic encephalopathy [10]. *Phoneutria nigriventer* spider venom causes blood-brain barrier permeability and induces the expression of TNF-a in Purkinje neurons in rats [48].

These studies suggest that the induction of TNF-a expression in neurons, including Purkinje neurons, may occur in different pathological situations and is preceded by TNF-a induction in glial cells. However, the underlying mechanisms remain unclear.

We show here that sustained hyperammonemia and peripheral inflammation induce TNF-a expression in Purkinje neurons in rats and in patients who die with chronic liver cirrhosis and hyperammonemia. Moreover, we provide some insights into the time course of and mechanisms involved in this process (Fig. 12). Hyperammonemia induces the activation of microglia and astrocytes at 1 week. This is followed by the induction of TNF-a in both types of glial cells (but not in Purkinje cells) at 2 weeks. TNF-a released by glial cells activates TNFR1 in Purkinje neurons, leading to the nuclear translocation of NF-κB and the induction of TNF-a expression.

The induction of TNF-a by sustained hyperammonemia may contribute to the neurological alterations observed in hyperammonemia and hepatic encephalopathy by altering the function of Purkinje neurons and neurotransmission and by inducing neuronal death.

The trafficking and surface membrane expression of AMPA and NMDA receptors is altered by high levels of TNF-a [49–54]. Increased TNF-a may alter the membrane expression and function of AMPA and NMDA receptors in Purkinje neurons, thus altering their activity and associated neurotransmission. This may contribute to impaired motor function in hyperammonemia and hepatic encephalopathy.

Moreover, TNF-a has been shown to induce excitotoxicity-mediated neuronal loss in some neurodegenerative diseases [55]. Under neuroinflammatory conditions, TNF-a induces glutamate release from microglia and astrocytes via TNFR1 stimulation, the induction of glutaminase and increased synthesis and release of glutamate. TNF-a also inhibits glutamate uptake in astrocytes, further increasing extracellular glutamate levels [54]. Furthermore, in neurons, TNF-a rapidly increases excitatory synaptic strength via TNFR1, inducing excessive calcium entry and producing excitotoxic neuronal death [54]. Increased TNF-a expression may also contribute to the degeneration of Purkinje neurons in the cerebellum of patients who die with liver cirrhosis, as reported recently by our group [20, 21].

## Conclusions

In summary, this study shows that sustained (but not short-term) hyperammonemia induces TNF-a expression in Purkinje neurons; this may be due to the activation of TNFR1 by TNF-a produced in glia (and later also by TNF-a produced in Purkinje cells), which induces the nuclear translocation of NF-κB and the induction of TNF-a mRNA transcription. The induction of TNF-a in Purkinje neurons alters the function of these cells and contributes to altered neurotransmission and neurological impairment. TNF-a is also induced in the Purkinje neurons of patients who die with liver cirrhosis and contributes to the loss of Purkinje cells in these patients. The induction of TNF-a in the Purkinje neurons of hyperammonemic rats is prevented in vivo by peripheral treatment with an anti-TNF-a antibody and ex vivo by treatment with R7050, which prevents the transduction of TNFR1 signals into Purkinje neurons, the nuclear translocation of NF-κB and the induction of TNF-a. This suggests that treatments that reduce peripheral inflammation or the activation of NF-κB may reduce the deleterious effects of sustained hyperammonemia and hepatic encephalopathy. The use of molecules to inhibit elements of the NF-κB signaling pathway has also been suggested as a therapeutic option for neurological disorders such as [56] multiple sclerosis, Parkinson’s disease [57], Alzheimer’s disease [58], and spontaneous intracerebral hemorrhage [38].

## Data Availability

All raw data used and analyzed in the current study are available from the corresponding author on reasonable request.

## Abbreviations

cIAP1: Inhibitor of apoptosis protein 1
cIAP2: Inhibitor of apoptosis protein 2
DAPI: 4′,6-Diamidino-2-phenylindole
DD: Death domain
GFAP: Glial fibrillary acidic protein
IBA-1: Ionized calcium-binding adaptor molecule 1
MHE: Minimal hepatic encephalopathy
RIP1: Receptor interacting protein 1
TNF-a: Tumor necrosis factor alpha
TRADD: TNFR-associated death domain
